# Heavy metal accumulation in and food safety of shark meat from Jeju island, Republic of Korea

**DOI:** 10.1371/journal.pone.0212410

**Published:** 2019-03-13

**Authors:** Sang Wha Kim, Se Jin Han, Yonggab Kim, Jin Woo Jun, Sib Sankar Giri, Cheng Chi, Saekil Yun, Hyoun Joong Kim, Sang Guen Kim, Jeong Woo Kang, Jun Kwon, Woo Taek Oh, Jehyun Cha, Seunghee Han, Byeong Chun Lee, Taesung Park, Byung Yeop Kim, Se Chang Park

**Affiliations:** 1 Laboratory of Aquatic Biomedicine, College of Veterinary Medicine and Research Institute for Veterinary Science, Seoul National University, Seoul, Republic of Korea; 2 Department of Statistics, College of Natural Sciences, Seoul National University, Seoul, Republic of Korea; 3 Department of Aquaculture, Korea National College of Agriculture and Fisheries, Jeonju, Republic of Korea; 4 Laboratory of Aquatic Nutrition and Ecology, College of Animal Science and Technology, Nanjing Agricultural University, Nanjing, China; 5 School of Mechanical Engineering, Hanyang University, Seoul, Republic of Korea; 6 School of Earth Sciences and Environmental Engineering, Gwangju Institute of Science and Technology, Gwangju, Republic of Korea; 7 Department of Theriogenology and Biotechnology, College of Veterinary Medicine, Seoul National University, Seoul, Republic of Korea; 8 Department of Marine Industry and Maritime Police, College of Ocean Science, Jeju National University, Jeju, Republic of Korea; Chinese Academy of Sciences, CHINA

## Abstract

Shark meat is consumed as a food source worldwide, especially in Asian countries. However, since sharks are apex predators in the ocean food chain, they are prone to bioaccumulation of heavy metals. More than 100 million sharks are caught annually for human consumption, and the safety of shark meat cannot be overemphasized. Here, we examined heavy metal concentration in the muscle tissue of 6 shark species including 3 migratory species (*Carcharhinus brachyurus*, *Carcharhinus obscurus*, and *Isurus oxyrinchus*) and 3 local species (*Triakis scyllium*, *Mustelus manazo*, and *Cephaloscyllium umbratile*) from fish markets in Jeju Island, Republic of Korea. The concentrations of 11 heavy metals (Cr, Fe, Cu, Zn, As, Se, Cd, Sn, Sb, Pb, and Hg) and MeHg were analyzed. The result showed that the average concentrations of all metals, except for that of As, were below the regulatory maximum limits of many organizations, including the Codex standard. Hg and MeHg were significantly correlated with body length, body weight, and age, and the concentration of Hg was expected to exceed the limit in *C*. *brachyurus* with a body length or weight of over 130 cm or 25 kg, respectively. Our results indicate that shark meat can expose consumers to a high level of As and that copper sharks bigger than the predicted size should be avoided for excessive Hg. Considering these findings, a detailed guideline on consumption of meat of different shark species should be suggested based on further investigation.

## Introduction

Shark meat has been used as a food source since the fourth century, and there was a drastic increase in its usage after World War I [1]. World shark capture production and shark meat trade amounts have increased continuously since 1950s, reaching the highest values on records in 2011 [2, 3]. Shark population thus has decreased due to unregulated shark fishing worldwide [4]. Currently, 28% of non-data deficient shark species are endangered according to the International Union for the Conservation of Nature (IUCN) red list [5]. Although the capture amount has decreased since 2011, the reason is still unclear whether there has been a decrease in demand or a decrease in population [6]. Asia is a major contributor to the global chondrichthyan market. Indeed, Japan, China, Taiwan, Hong Kong, Singapore, Republic of Korea, Thailand, India, Indonesia, and Malaysia are all involved in the production, import, and export of shark meat [1]. Asian countries accounted for 59% of the global shark trade from 1976 to 2015; this percentage has increased to 63% in the last 15 years [3].

In the Republic of Korea, ancient records on shark products as food sources or clinical medicines include Shinjeung-donggukyeojiseungram (新增東國輿地勝覽, Augmented Geographical Survey of Korea, 1481), Donguibogam (東醫寶鑑, Principles and Practices of Eastern Medicine, 1613), Jangjeon Ganchal (張瑱 簡札, Jangjeon’s letter, 1690), and Jasaneobo (玆山魚譜, Record of Fish in Heuksan Island, 1814), and shark meat continues to be a major food source at present. The amount of shark meat imported into Korea surged in the 1990s and has remained steady at over 20,000 tons since 2003. Moreover, the import value has increased, even after 2003, and was reported to be 85 million USD in 2015 [3]. This increase in demand can be explained by the routine intake of shark meat in Korea, both as a traditional dish in Gyeongsangbuk-Do province and as a general cuisine in the form of steamed meat, roasted meat, soup, and skewers in various provinces.

Fish meat consumption has various advantages, including reducing the risk of cardiovascular disease and osteoporosis and facilitating the development of embryos via increased intake of long-chain n-3 fatty acids [7]. However, various environmental toxins including dioxin, polychlorinated biphenyls, and heavy metals can also be introduced into the body by consuming fish meat. Toxins can accumulate in the fish body via pollution sources such as industrial waste waters from mine and refinery [8–10]. Among various toxins, heavy metal accumulation is a critical parameter for establishing food safety of shark meat because of the multiple harmful effects of heavy metals. Fish meat is the main source of mercury (Hg), arsenic (As), copper (Cu), selenium (Se), lead (Pb), and cadmium (Cd) in diet. Many national and international organizations have developed regulatory maximum limits for different heavy metals to avoid damage to the body by fish consumption [11].

Shark is a representative apex predator of marine ecosystems and its meat has a higher risk of bioaccumulation of environmental toxins than other fish species [12]. Since shark meat has been used as an important food source for a long time, a large number of studies have been conducted on heavy metal concentration to ensure food safety [8, 9, 12–21]. The concentrations of various heavy metals have been measured at the level of species, habitat, environmental conditions, and biological conditions (age, body length, body weight, etc.). Based on the accumulated information, the food safety of shark meat is being assessed.

Among various heavy metal species, methylmercury (MeHg) especially shows a good absorption rate (90%) and long retention time (half-life: 70 days) in the human body. Excessive accumulation of MeHg can induce atrophy of the cerebral cortex, ataxia, hearing loss, decreased visual acuity, and increased incidence of cardiovascular diseases. The main source of MeHg in humans is fish meat intake because MeHg is biomagnified through the marine and freshwater food chain. Indeed, terrestrial animals typically have about 20 μg/kg MeHg, whereas large fish species, such as tuna or sharks, can have MeHg concentrations of up to 1 mg/kg [7, 22]. MeHg concentrations in fish depend on many factors, including the concentrations of water and sediments, the pH and redox potential of water, and the age, species, and size of fish [23]. Therefore, further studies are necessary to ensure the safety of fish meat consumption, especially meat from apex predators.

*Carcharhinus brachyurus* (copper shark), making up 60% of the shark species used in this study, are pelagic, oceanic, and highly migratory species that are distributed all over the coastal areas of tropical and temperate seas. They have been fished commercially in many countries worldwide including New Zealand, Australia, South Africa, Brazil, Uruguay, Argentina, Mexico, China, and Korea [24, 25]. They migrate to the north in the spring and summer and migrate back to the south in the fall and winter, reaching the sea around Jeju island from October to February. These sharks consume cephalopods, pelagic and benthic teleosts, and small elasmobranchs as their main food, staying at the top of the ocean food chain and are by-caught in the Jeju island mainly with Japanese amberjack (*Seriola quinqueradiata*), longtooth grouper (*Epinephelus bruneus*), and largehead hairtail (*Trichiurus lepturus*) [25, 26]. They are sold at wholesale fish markets, transported to other areas, and consumed by local people. Since they are being caught and consumed not only in Korea but also in all countries, the food safety of copper shark meat should be a concern. As they are the apex predators of the ocean food chain, the need for bioaccumulation analysis is particularly emphasized.

Accordingly, in this study, we evaluated the concentrations of heavy metals (Cr, Fe, Cu, Zn, As, Se, Cd, Sn, Sb, Pb, Hg, and MeHg) in six shark species including *C*. *brachyurus* captured at Jeju island (*C*. *brachyurus*, *Carcharhinus obscurus*, *Isurus oxyrinchus*, *Triakis scyllium*, *Mustelus manazo*, and *Cephaloscyllium umbratile*). We also examined correlations between metal concentrations and biological criteria including age, sex, total body length (TBL), body weight (BW), and habitat in *C*. *brachyurus*. With these results, we evaluated the safety of consuming the meat of these six shark species.

## Materials and methods

### Sampling

**Fig 1:**
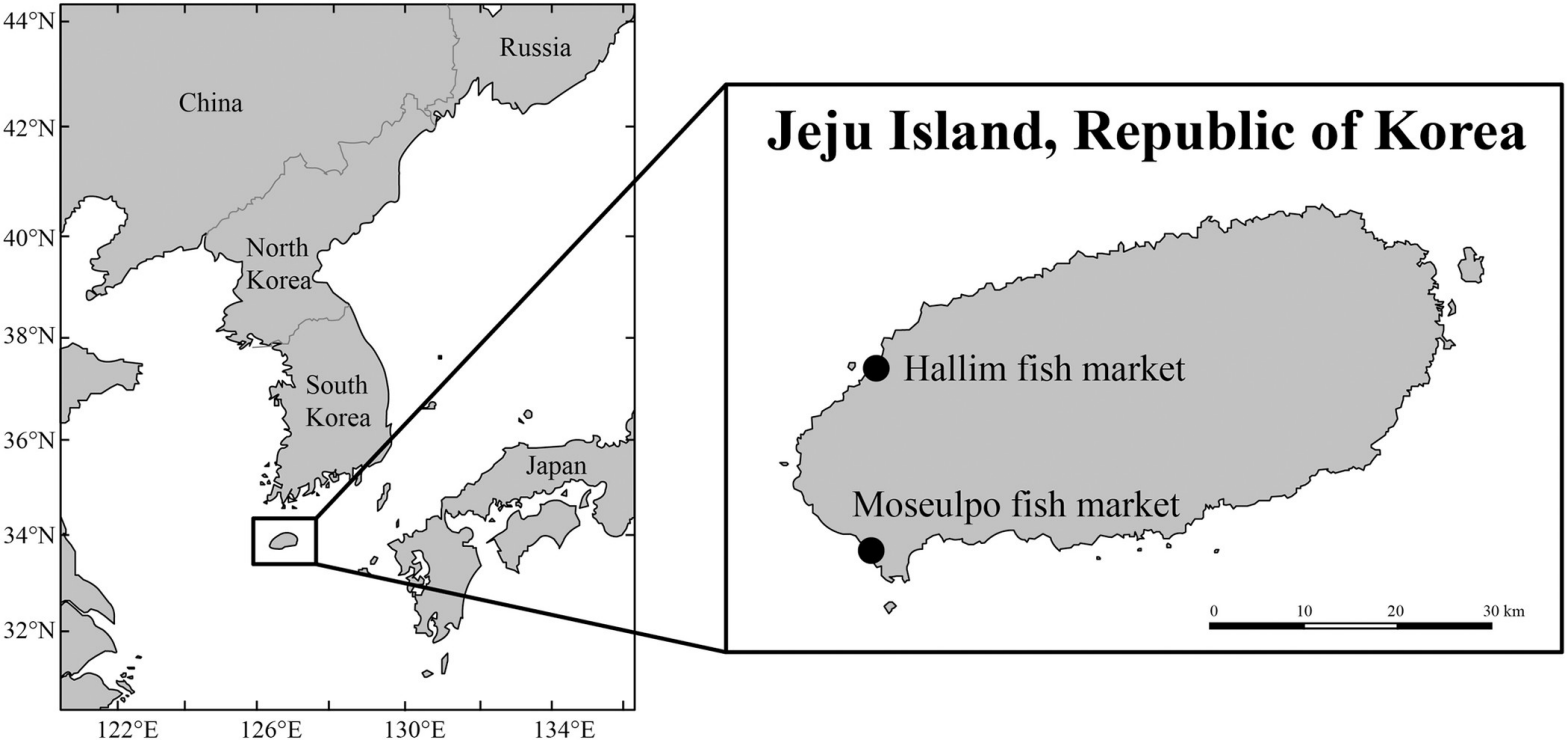
Sampling location in Jeju Island. Nineteen sharks were sampled from the Moseulpo fish market (30, Hamohanggu-ro, Daejeong-eup, Seogwipo-si, Jeju-do 63506, Republic of Korea) and 6 sharks were from the Hallim fish market (141–3, Hallimhaean-ro, Hallim-eup, Jeju-si, Jeju-do 63032, Republic of Korea).

Sharks were sampled at Moseulpo and Hallim fish markets in Jeju, Korea from October 2017 to December 2017, immediately after being released to the markets from fishing ships, targeting the ‘shark meats actually being sold at the fish markets’ as our sample population (Fig 1). For the Moseulpo fish market, a total of 19 sharks were by-caught from the sea area between Gapa island and Mara island by line fishing ships for Japanese amberjack (*Seriola quinqueradiata*), angling fishing ships for largehead hairtail (*Trichiurus lepturus*), and longline fishing ships for longtooth grouper (*Epinephelus bruneus*). All these 19 sharks were sampled immediately after being auctioned. For the Hallim fish market, sharks were by-caught by drift gillnets, and six of these sharks were sampled. Almost every shark brought to the markets during the sampling period was sampled to obtain the shark meat population actually consumed by the local people. All samples were numbered from SNU-MO-0001 to SNU-MO-0025 in chronological order. The species of the sharks were determined with their features based on previous studies [27, 28]. Sharks were sampled and necropsied to determine their health statuses within 12 h of being caught. TBL, BW, and the girth right in front of the first dorsal fin were measured. The epaxial muscle and 4–6 vertebrae were sampled at the level of the first dorsal fin. Muscles were sampled using a Whirl-Pak (Sigma-Aldrich, Germany) and kept frozen at -20°C until analysis. Muscle tissues on the vertebrae were roughly removed, and all vertebral joints were disparted. The neural arch and transverse processes were removed from the centrums. Remnant tissue was removed after the centrums were immersed in commercial sodium hydroxide solution. Then the centrums were rinsed with water and dried for more than 72 h.

### Age determination

Completely dried centrums were embedded with EPOXY Resin (Pace Technologies, AZ, USA) and EPOXY Hardener (Pace Technologies). After 24 h of hardening, centrums were sectioned using an Isomet low-speed saw (Buehler, IL, USA) with a diamond saw into 2-mm-thick pieces. Samples were then attached to slide glasses using 7036.20 Blanchard Wax (J.H. Young Company, NY, USA), and then the centrum pieces were ground using an AutoMet 250 grinder-polisher (Buehler) with sandpaper until the slices were thinner than 200 μm. Samples were ground in two stages using different grinding papers (Carbimet 320 grit and 600 grit) to minimize the surface scratch. The annulus on the corpus calcareum was measured on the slides independently by three researchers.

### Heavy metal concentration analysis

#### Evaluation of total heavy metals by inductively coupled mass spectrometry (ICP-MS)

Total concentrations of 10 heavy metals (chromium [Cr], iron [Fe], copper [Cu], zinc [Zn], arsenic [As], selenium [Se], cadmium [Cd], tin [Sn], antimony [Sb], and lead [Pb]) were analyzed using ICP-MS. Approximately 0.1 g of homogenized shark muscle in wet condition was completely disintegrated by adding 10 mL HNO_3_ and heating at 200°C for 2 h. Samples were cooled to room temperature, deionized water was added to the digested samples, and the dilution factors were calculated by measuring the final weight. ICP-MS was conducted with an Agilent 770x ICP-MS (Agilent Technologies, CA, USA). Analysis was carried out in He mode with a low matrix concentration, and the CeO/Ce ratio was 0.9%. Certified reference materials (CRMs; VHG Labs, NH, USA) were used at a concentration of 0.1–100 ppb for calibration (Pearson’s r > 0.999). Analysis results were presented in mg/kg, and the detection limit (DL) was 0.0001 mg/kg (KOPTRI, Seoul, Korea).

#### Total mercury analysis

Shark muscle tissues were cut into 1-cm^3^ pieces and freeze-dried for 72 h using a freeze-dryer (FDU-1200; Eyela Co., Japan). The moisture composition ratio was calculated using the mass difference between before and after freeze-drying. Freeze-dried samples were homogenized thoroughly using a mortar, placed into conical tubes, and sealed with parafilm until use. For total mercury analysis, approximately 0.01 g of freeze-dried and homogenized shark muscle samples was weighed on a precision scale connected to a Milestone DMA-80 Direct Mercury Analyzer (Milestone, Bergamo, Italy) and analyzed. DOLT-5 (National Research Council Canada, Ottawa Ontario, Canada) was used as a CRM, and the average recovery of CRM was 103.7% (± 9.8%, n = 13). The DL measured by DMA-80 was 0.06 ng/g. Dry weight concentrations were converted into wet weight concentrations using the moisture composition ratio.

#### Methyl mercury analysis

For methylmercury analysis, approximately 0.01 g freeze-dried and homogenized shark muscle sample and DOLT-5 CRM were weighed on a precision scale and placed in glass bottles that had been washed with 15% HCl (12 N) solution. After adding 5 mL of 25% KOH solution diluted with methanol, samples were incubated in an oven at 60°C over 12 h. Samples were cooled to room temperature, and deionized water was added to the sample solution for dilution. Next, 200 mL of deionized water was added to the bubbler, and 1 mL acetate buffer (2.0 M) was added to adjust the pH to 4.9. Diluted sample solution and 0.1 mL of 1% tetraethylborate solution were added to the bubbler to produce volatile methylethylmercury. Volatile methylethylmercury was collected into a Tenax trap after 15 min of N_2_ gas purging and 15 min of incubation for ethylation to methylated Hg species. The traps were then dried with N_2_ gas for 9 min. Hg species were released from the Tenax trap following 30 s of heating, separated into individual species using gas chromatography (GC), and detected by cold vapor atomic fluorescence spectroscopy (CVAFS) using a Brooks Rand Model III detector (Brooks Rand Instruments, WA, USA). The average recovery rate of CRM was 101.6% (± 8.1%, n = 9). Dry weight concentrations were converted to wet weight concentrations using the moisture composition ratio.

#### Statistical analysis

When non-detects that have a lower concentration than that of DL comprise less than 15% of the data, replacement with 1/2DL is suggested as one of the satisfactory methods for further analysis [29, 30], and based on this suggestion, many papers have used the 1/2DL substitution method for statistical analysis of data with non-detects [31–33]. Therefore, for a more detailed statistical analysis other than simple average comparison, 1/2DL was used as a substitute for non-detects, and only the heavy metals in which the non-detect portion was lower than 15% were analyzed (Fe, Cu, Zn, As, Se, Hg, and MeHg). In cases of biological data acquisition failure due to field situations, the related data were excluded from statistical processing.

Multivariate analysis of variance (MANOVA) was used to determine whether species, sex, and habitat affect heavy metal concentrations. Since MANOVA analysis assumes both multivariate normality and homogeneity of variance-covariance matrix of residuals and the raw data did not satisfy the assumptions (S1 Table), multivariate Box-Cox transformation was performed using estimated transformation parameters (S2 Table) [34]. Since the transformation was applied to practical situations, estimated power parameters were replaced by simple power parameters, i.e., 0, ±0.25, and ±0.5. The Box-Cox family requires the responses to be strictly positive. Transformation was performed using the powerTransform function in Car version 3.0–2 package in R [35]. *λ* is the power parameter in the following equation.

$$y(\lambda )=\{\begin{array}{ll} \frac{y^{\lambda }-1}{\lambda } & if\;\lambda \neq 0\\ \log(y) & if\;\lambda =0 \end{array}$$

Shapiro-Wilk test for marginally normality test, Bartlett’s test for homogeneity of variance-covariance matrix assumption check, Mardia’s multivariate normality (MVN) test, and Henze-Zirkler’s MVN test for jointly normality tests were performed using statistical package R [35]. MVN tests were performed with MVN package version 5.5 in R [36]. Normal Q-Q plots were also generated to check normality. Since the assumptions were satisfactory after proper transformation (S3 and S4 Tables), MANOVA was conducted with car package version 3.0–2 in R. Data of *C*. *umbratile* was excluded when MANOVA was performed for species due to the small sample number (sample number was only one).

Multivariate regression (MVR) was also performed to confirm whether there is any significant correlation between age, BW, girth, TBL and each heavy metal concentration using the lm function in R. To satisfy the fundamental assumptions of multivariate linear regression, the raw data were transformed using multivariate Box-Cox transformation for each covariate: age, BW, TBL, and girth. All responsible variables were tested with Shapiro-Wilk test, Bartlett’s test, Mardia’s MVN test, and Henze-Zirkler’s MVN test, and we confirmed that they satisfy the assumptions.

To elucidate the correlations among parameters, excluding species differences, MANOVA and MVR were conducted using data from *C*. *brachyurus* only. Pearson’s coefficients and p values were also calculated using R to confirm statistical significance [35]. Based on the results from MVR showing significant correlation between heavy metal concentration and biological parameters of *C*. *brachyurus*, linear and polynomial regressions were used and compared to each other to identify a more, well-fitting model using OriginPro 8.5 (OriginLab Corporation, Northampton, USA).

## Results

### Characteristics of the sampled sharks

In total, 25 sharks of six species were collected over a 3-month period. *C*. *brachyurus* (copper shark), *C*. *obscurus* (dusky shark), and *I*. *oxyrinchus* (shortfin mako) were by-caught by line fishing, angling fishing, and longline fishing in the Moseulpo sea, and *T*. *scyllium* (banded houndshark), *M*. *manazo* (starspotted smooth-hound), and *C*. *umbratile* (blotchy swell shark) were by-caught by drift gillnets in the Hallim sea area. We could not collect some of the biological data due to circumstantial causes. Collected biological data are summarized in Table 1 by species, and all the raw data are recorded in the S5 Table. Because sharks longer than 2 m are difficult to draw back to land when by-caught, sharks distributed in fish markets are generally less than 2 m in length. Indeed, the sharks collected for this investigation were all shorter than 2 m. Thus, for shark species that often have mature lengths of over 2 m, including *C*. *brachyurus*, *C*. *obscurus*, and *I*. *oxyrinchus*, only juvenile sharks were collected. As a result of age estimation using vertebral section, all sharks were found to be younger than 7 years old (Table 1). Based on this information, the sampled batch was not a true representation of *C*. *brachyurus*, *C*. *obscurus*, and *I*. *oxyrinchus*. However, since the collected specimens were subsamples of sharks that are actually consumed by the local people, this batch was a good representation of shark meat as a food material, which is the target sample population of this research. Moreover, since these three species (*C*. *brachyurus*, *C*. *obscurus*, and *I*. *oxyrinchus*) show seasonal migration and can be consumed in other countries on their migration route, the importance of their food safety becomes greater.

**Table 1:** Total body length, body weight, age, and sampling location of sampled sharks.

Species	*n*	Mean ± SD (Min-Max)			Sampling location
		Total body length (cm)	Body weight (kg)	Age (yr)	
*Carcharhinus brachyurus*	15	124.02 ± 32.70 (68–190)	15.33 ± 12.06 (4.5–45)	2.64 ± 1.86 (0–7)	Moseulpo fish market
*Carcharhinus obscurus*	2	116.00 ± 2.83 (114–118)	8.45 ± 0.78 (7.9–9)	0.0 ± 0.0 (0–0)	Moseulpo fish market
*Isurus oxyrinchus*	2	123.50 ± 19.09 (110–137)	12.60 ± 7.21 (7.5–17.7)	1.0 ± 0.0 (1–1)	Moseulpo fish market
*Triakis scyllium*	3	70.00 ± 3.77 (66.5–74)	1.70 ± 0.00 (1.7–1.7)	2.0 ± 0.0 (2–2)	Hallim fish market
*Mustelus manazo*	2	68.00 ± 22.63 (84–52)	0.50 ± 0.00 (0.5–0.5)	2.0 ± 0.0 (2–2)	Hallim fish market
*Cephaloscyllium umbratile*	1	63.0	-	-	Hallim fish market

Data not available were excluded from the mean and standard deviation calculation. Missing value proportions of total body length, body weight, and age are 4%, 32%, and 24%, respectively. Sharks on the first year after birth were counted as 0 years old. All average and standard deviation values were rounded to the second decimal place.

### Average heavy metal concentrations

The average moisture composition ratio was 74.6%, and the dry weight concentrations were converted to wet weight concentration using this value. The average concentrations of the 11 metals and MeHg in all 25 sharks are indicated in Table 2 and showed the following decreasing order: Fe > As > Zn > Cu > Se > Hg > MeHg > Cr > Pb > Sn > Sb > Cd. The average concentrations of Cr, Sn, and Pb were the highest in *C*. *obscurus*, whereas the average concentrations of Fe, Cu, and Hg were the highest in *I*. *oxyrinchus*. As and Se concentrations were the highest in *M*. *manazo* and *T*. *scyllium*, respectively. Zn and Sb were the highest in *C*. *umbratile*. For Hg, *I*. *oxyrinchus* had the highest concentration (0.27 mg/kg) followed by *C*. *obscurus*, *C*. *brachyurus*, *C*. *umbratile*, *T*. *scyllium*, and *M*. *manazo*. The Cd concentration was under the DL (0.0001 mg/kg) in all 25 sharks, similar to previous studies showing that most of the absorbed Cd were stored in the liver and that only a small amount was stored in the muscle in sharks [12, 13].

**Table 2:** Average concentrations (mg/kg wet weight) of 11 metals and methyl mercury in six shark species.

Species (scientific name)	*n*	Metal concentration (Mean ± SD)											
		Cr	Fe	Cu	Zn	As	Se	Cd	Sn	Sb	Pb	Hg	MeHg
*Carcharhinus brachyurus*	15	0.07 ± 0.15	8.22 ± 7.54	1.39 ± 2.25	7.24 ± 5.62	6.98 ± 3.29	0.52 ± 0.79	0.00 ± 0.00	0.03 ± 0.05	0.01 ± 0.02	0.04 ± 0.05	0.25 ± 0.25	0.16 ± 0.21
*Carcharhinus obscurus*	2	0.37 ± 0.53	12.63 ± 14.68	2.73 ± 3.67	8.16 ± 4.01	7.85 ± 0.24	0.35 ± 0.04	0.00 ± 0.00	0.09 ± 0.13	0.00 ± 0.00	0.08 ± 0.06	0.26 ± 0.02	0.17 ± 0.00
*Isurus oxyrinchus*	2	0.00 ± 0.00	13.48 ± 1.78	4.45 ± 4.67	3.23 ± 1.60	2.06 ± 1.07	0.36 ± 0.09	0.00 ± 0.00	0.07 ± 0.08	0.00 ± 0.00	0.06 ± 0.02	0.27 ± 0.07	0.19 ± 0.08
*Triakis scyllium*	3	0.31 ± 0.40	9.97 ± 13.48	0.18 ± 0.08	3.41 ± 0.62	8.32 ± 0.37	0.64 ± 0.17	0.00 ± 0.00	0.00 ± 0.00	0.00 ± 0.00	0.02 ± 0.01	0.13 ± 0.02	0.07 ± 0.02
*Mustelus manazo*	2	0.07 ± 0.08	2.03 ± 2.87	0.43 ± 0.35	2.09 ± 0.08	17.59 ± 3.63	0.41 ± 0.05	0.00 ± 0.00	0.00 ± 0.00	0.00 ± 0.00	0.01 ± 0.01	0.12 ± 0.04	0.08 ± 0.02
*Cephaloscyllium umbratile*	1	0.16	5.66	0.57	13.56	8.09	0.24	0.00	0.00	0.08	0.07	0.18	0.11
Total	25	0.12 ± 0.23	8.60 ± 8.08	1.49 ± 2.37	6.37 ± 5.06	7.71 ± 4.25	0.49 ± 0.62	0.00 ± 0.00	0.03 ± 0.06	0.01 ± 0.02	0.04 ± 0.05	0.21 ± 0.20	0.13 ± 0.16

Values under DL were substituted with half the value for statistical analysis. All values were rounded to the second decimal place. SD: standard deviation.

### Correlations between heavy metal concentrations and biological information

After the Box-Cox transformation, all the data satisfied the multivariate normality assumption and homogeneity of variance-covariance matrix assumption, except for only 6 cases (S3 Table). Even though a few samples tested non-normal or had a slight inequality of variance, MANOVA test could be performed since it is fairly robust for deviations from multivariate normality and homogeneity of variance-covariance.

MANOVA of Fe, Cu, Zn, As, Se, Hg, and MeHg concentrations for the 25 sharks showed that the concentrations of these heavy metals were significantly associated with species (multivariate significance = 0.024; Table 3). Specifically, Zn and As concentrations were significantly correlated with species.

**Table 3:** MANOVA test.

Variables	MVS	Metals						
		Fe	Cu	Zn	As	Se	Hg	MeHg
***All sharks***								
Species	0.024*	0.346	0.261	0.045*	0.001***	0.740	0.471	0.452
Sex	0.909	0.933	0.372	0.216	0.762	0.689	0.582	0.581
Habitat	0.240	0.459	0.208	0.234	0.016*	0.474	0.313	0.451
***Carcharhinus brachyurus***								
Sex	0.289	0.264	0.395	0.452	0.069	0.489	0.334	0.337

Multivariate significance (MVS) was calculated by analyzing Fe, Cu, Zn, As, Se, Hg, and MeHg based on the criteria of species, sex, and habitat of sharks. For *C*. *brachyurus*, MANOVA could be performed by incorporating sex. All values were rounded to the third decimal place.

***: *p* < 0.001

**: *p* < 0.01

*: *p* < 0.05.

**Fig 2:**
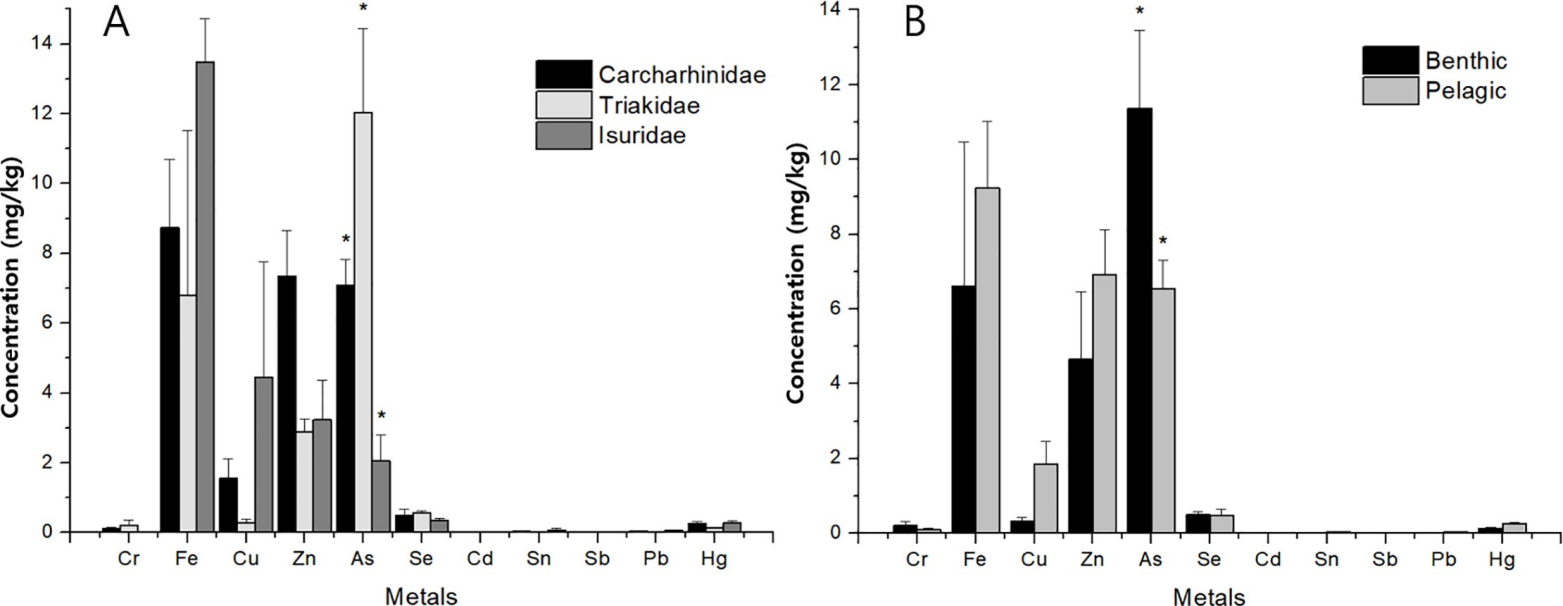
Average heavy metal concentration comparison by family and habitat. Data under the DL were substituted with half the DL. Error bars indicate standard errors. *: p < 0.05; Pelagic sharks: *C*. *brachyurus*, *C*. *obscurus*, *I*. *oxyrinchus*; benthic sharks: *T*. *scyllium*, *M*. *manazo*, *C*. *umbratile*. Carcharhinidae: *C*. *brachyurus*, *C*. *obscurus*; Triakidae: *T*. *scyllium*, *M*. *manazo*; Isuridae: *I*. *oxyrinchus*.

In the case of As, it showed a significant correlation not only with species but also with habitat and family (Fig 2A and 2B). The concentration of As differed between benthic and pelagic sharks according to habitat-based classification. *T*. *scyllium*, *M*. *manazo*, and *C*. *umbratile* are benthic sharks, whereas *C*. *brachyurus*, *C*. *obscurus*, and *I*. *oxyrinchus* are pelagic sharks (Fig 2B). As concentrations were significantly higher in benthic sharks (p < 0.05), and the pattern was similar to that of a previous study showing higher concentrations of As in bottom-feeding fish than in other bony fish [22]. Difference in As concentration was also found according to phylogenetic families, i.e., Carcharhinidae (*C*. *brachyurus* and *C*. *obscurus*), Isuridae (*I*. *oxyrinchus*), and Triakidae (*T*. *scyllium* and *M*. *manazo*; Fig 2A), presenting significantly different concentrations (p < 0.05) among the three groups. In particular, As was the highest in Triakidae. Since Triakidae sharks studied in this study are all benthic sharks, these results can be interpreted in the same context.

In the case of Zn, the concentration was the highest in Carcharhinidae sharks (Fig 2A). The concentration decreased in the order of Carcharhinidae, Isuridae, and Triakidae, and this resembles the pattern of Zn concentration appearing higher in pelagic sharks than in benthic sharks. This is also due to the fact that Carcharhinidae and Isuridae are pelagic sharks.

Other heavy metals also showed concentration differences according to habitat. In the case of Hg, the average concentration in benthic sharks was 0.14 mg/kg, which was 44% lower than that in pelagic sharks (0.25 mg/kg). The MeHg concentration in benthic sharks (0.08 mg/kg) was 53% lower than that in pelagic sharks (0.17 mg/kg). This is consistent with a previous study showing different Hg and MeHg accumulation pattern between pelagic and benthic food chains [37]. Additionally, the average concentrations of Cu, Zn, Sn, and Pb were higher in pelagic sharks, whereas the average concentrations of Fe and As were higher in benthic sharks.

In the MVR analysis to confirm the correlation between continuous variables (age, BW, girth, and TBL) and heavy metal concentrations, Hg and MeHg were found to be significantly and positively correlated with BW, girth, and TBL (Table 4, S1 Fig). A significant positive correlation also existed between Zn and girth, Fe and girth, and Fe and TBL. The correlation between Zn and girth is comparable with that of a previous study; as in the case of the teleost fish *Poecilia reticulata*, it is known that Zn concentration is maintained at a certain level in the body of these fish [38].

**Table 4:** Multivariate regression (MVR).

Variables	Fe	Cu	Zn	As	Se	Hg	MeHg
***All sharks***							
Age	0.681	0.522	0.912	0.932	0.058	0.374	0.653
BW	0.400	0.966	0.617	0.435	0.140	0.001**	0.002**
Girth	0.032*	0.166	0.031*	0.091	0.029*	0.008**	0.017*
TBL	0.033*	0.478	0.276	0.151	0.043*	0.001***	0.001**
***Carcharhinus brachyurus***							
Age	0.384	0.837	0.954	0.718	0.123	0.122	0.230
BW	0.554	0.571	0.879	0.721	0.173	0.002**	0.005**
Girth	0.079	0.245	0.828	0.702	0.071	0.074	0.131
TBL	0.092	0.937	0.333	0.707	0.160	0.009**	0.011*

MVR was analyzed and p-values were calculated on Fe, Cu, Zn, As, Se, Hg, and MeHg on the criteria of age, BW, girth, and TBL. For *C*. *brachyurus*, MVR was also performed by incorporating the same biological criteria. All values were rounded to the third decimal place.

***: *p* < 0.001

**: *p* < 0.01

*: *p* < 0.05.

Se showed a significant correlation with girth and TBL, and was the only heavy metal that showed a negative correlation (S1 Fig). This pattern is different from those of previous studies showing that Se has positive correlation with TBL in some tropical fishes or even that Se does not have any correlation with TBL in various fish species including shortfin mako [39, 40].

### Correlations between heavy metal concentrations and biological information in *C*. *brachyurus*

**Fig 3:**
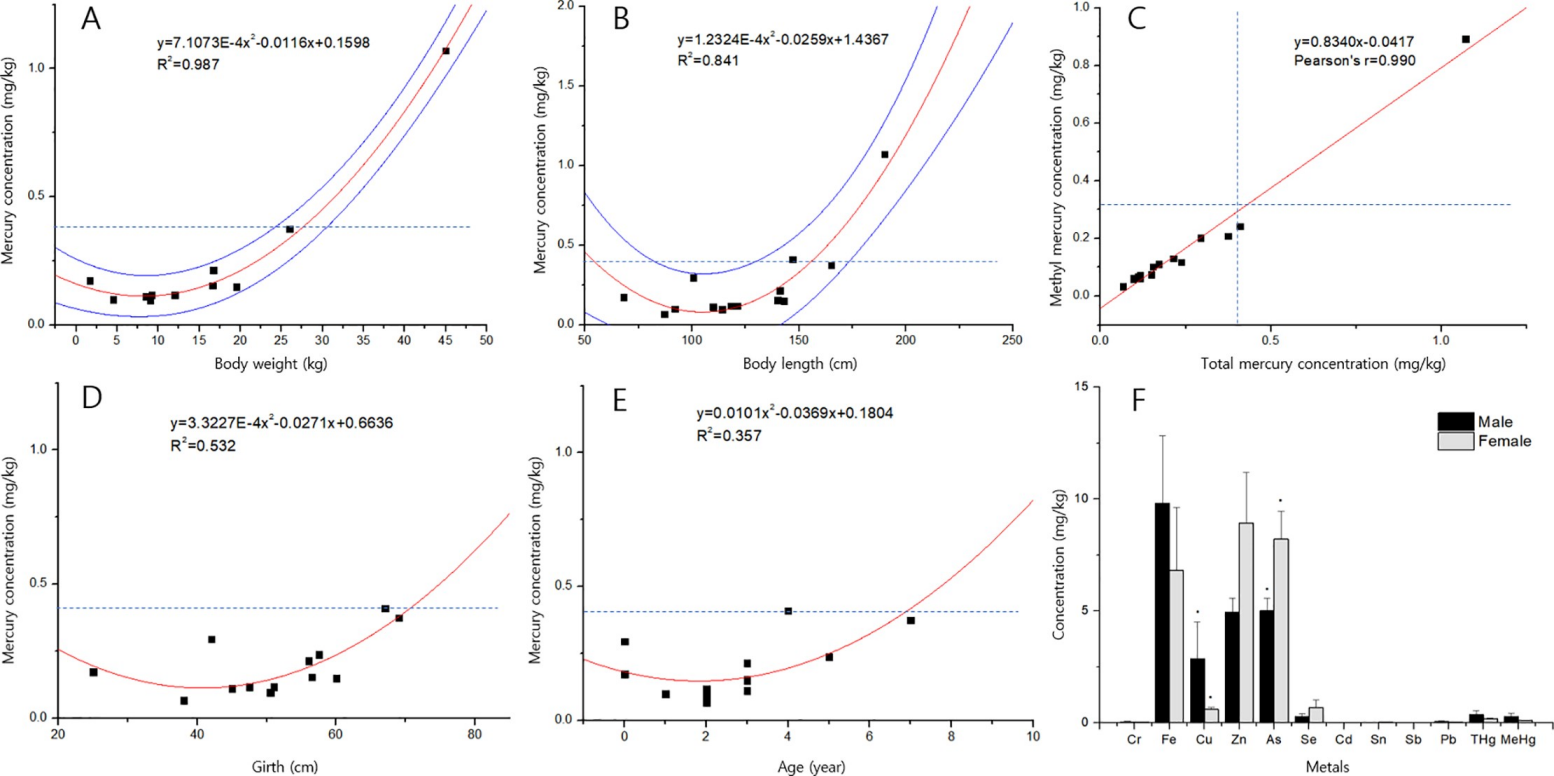
Heavy metal concentration analysis in *C*. *brachyurus*. A, B, D, E: Correlation between Hg concentration and body weight (BW), total body length (TBL), girth, and age in *C*. *brachyurus* (p < 0.05 in all four cases). Blue lines show the 95% prediction interval for the regression line, and blue dotted lines indicate the regulatory maximum limits of Hg and MeHg (0.4 and 0.3 mg/kg, respectively). C: Correlation between the concentrations of Hg and MeHg in *C*. *brachyurus* (Pearson’s r = 0.990, p < 0.05). F: Average heavy metal concentration comparison between male and female *C*. *brachyurus*. Error bars indicate standard errors, and the spots beyond the error bars indicate significance at p < 0.1.

The results of MANOVA in *C*. *brachyurus* (n = 15) showed that BW and TBL are significantly correlated with both Hg and MeHg (p < 0.05; Table 3, S1 Fig). To find a regression model that explains the correlations better, adjusted R^2^ values of polynomial and linear regressions were compared, and polynomial regression was concluded as a more, well-fitting model (Fig 3A and 3B). According to this regression model, Hg concentration in this species decreases from birth to a certain point in their life, and then increases with time. This model gives strength to the existence of growth dilution of Hg in *C*. *brachyurus*. The slight decrease in Hg concentration at the early stage of growth can be deduced from the effect of growth dilution in young individuals [17, 41]. Polynomial regression of Hg concentrations according to girth and age also demonstrated positive relationships (Fig 3D and 3E).

Based on this information, we speculated that Hg and MeHg concentrations were all under the regulatory maximum limits because of the sampled sharks’ ages and sizes. Prediction intervals of the regulatory maximum limit of Hg (0.4 mg/kg) calculated using the regression lines of BW and TBL were as follows: BW, 25.3–31.4 kg; TBL, 130.8–174.0 kg. These two criteria are easy to confirm in the field and can be checked before a shark is sold at a local market in order to avoid consumption of shark meat having Hg concentrations over the limit value.

Hg and MeHg concentrations showed strong positive linear correlations (Pearson’s r = 0.990; Fig 3C), and the average MeHg/Hg ratio was 61.5% ± 8.3% (min–max: 46.3–83.3%), similar to previous reports [18, 19]. *I*. *oxyrinchus* had the highest MeHg/Hg ratio (66.6%), and *T*. *scyllium* had the lowest MeHg/Hg ratio (55.8%).

Heavy metal concentration differences according to sex in *C*. *brachyurus* were analyzed. Fe, Cu, and Hg were high in males, whereas Zn, As, and Se were high in females. In particular, Cu and As showed significant differences (p < 0.1; Fig 3F). It is known that Cu concentration in some teleost fish is higher in females than in males due to the metabolic activity difference, and a similar mechanism can be considered for *C*. *brachyurus* in further studies [42].

By taking a closer look at the Cu concentration values, SNU-MO-0012 was a particularly noticeable case. This shark was by-caught right after birth, having the umbilical cord still attached and its intestine filled with meconium only. This shark’s Cu concentration was obviously the highest among all *C*. *brachyurus*, and the cumulative percentage was 99.9%. Although the reason for this high Cu concentration in younger individuals is unknown, this phenomenon is similar to that observed in humans. Indeed, human babies show peak Cu concentrations right after birth, with a subsequent decrease to one-tenth the initial level beginning after 13 weeks of age [43, 44]. Consistent with this, the Cu concentration in SNU-MO-0012 (8.91 mg/kg) was almost 10 times that of the other 14 *C*. *brachyurus* sharks (0.92 mg/kg). This tendency was consistent with a previous study showing that Cu concentration decreases during a shark’s growth period and increases again after their maturation [8, 45]. A similar tendency was also found in marine mammals whose Cu concentrations decreased with their growth [46, 47].

Pb concentrations showed a positive linear relationship with weight (Pearson’s r = 0.729, p < 0.05), as confirmed by linear regression analysis. A positive relationship was also observed for all 25 sharks (Pearson’s r = 0.578, p < 0.05), in contrast to previous studies demonstrating that there was a negative relationship between fish body weight or size and Pb concentration [48, 49].

The correlation matrix of the 11 heavy metals in *C*. *brachyurus* showed significant relationships between Fe and Cr (Pearson’s r = 0.538, p < 0.01), Sn and Pb (Pearson’s r = 0.437, p < 0.05), and Hg and Pb (Pearson’s r = 0.752, p < 0.001). However, the reason for these correlations is still unknown. In contrast, unlike the known positive correlation between Se and Hg concentration in fish muscle, Se and Hg concentrations in *C*. *brachyurus* were not significantly correlated (Pearson’s r = 0.04) [14, 50]. This pattern did not change when the correlation was analyzed with all the 25 sharks collected (Pearson’s r = 0.02).

## Discussion

### Regulatory limit of heavy metal concentration

Heavy metal concentration is one of the important criteria that should be confirmed for food safety. Various institutions and governments have suggested regulatory maximum limits for different heavy metals in fish (or predatory fish) meat. To confirm the safety of shark meat as a food product, heavy metal concentrations from this study were compared with the regulatory maximum limits from eight international regulatory bodies and one domestic (Korean) food regulatory body (Table 5).

**Table 5:** Regulatory maximum limits of metals in shark meat.

Species	Metal concentration comparison with regulatory maximum limits										
	Cr	Cu	Zn	As	Se	Cd	Sn	Sb	Pb	Hg	MeHg
Regulatory maximum limits (As, Cd, Sn, Sb, Pb, Hg, MeHg: mg/kg) (Cr, Cu, Zn, Se: mg/day)	0.25^d^	5^d^	25^d^	3^b^	0.3^d^	0.05~ 1^b,e,f,g,h^	50^b^	0.15^b^	0.2~ 2^a,b,c,e,f,g,h^	0.4~ 1^e,f,g,i^	0.3~ 1^a,b,c,h,i^
*Carcharhinus brachyurus*	0.01	0.12	0.51	6.98	0.04	0.00	0.03	0.01	0.04	0.25	0.16
*Carcharhinus obscurus*	0.03	0.22	0.65	7.85	0.03	0.00	0.09	0.00	0.08	0.26	0.17
*Isurus oxyrinchus*	0.00	0.35	0.26	2.06	0.03	0.00	0.07	0.00	0.06	0.27	0.19
*Triakis scyllium*	0.02	0.01	0.27	8.32	0.05	0.00	0.00	0.00	0.02	0.13	0.07
*Mustelus manazo*	0.01	0.03	0.17	17.59	0.03	0.00	0.00	0.00	0.01	0.12	0.08
*Cephaloscyllium umbratile*	0.01	0.05	1.08	8.09	0.02	0.00	0.00	0.08	0.07	0.18	0.11

The regulatory maximum limits and mean heavy metal concentrations of As, Cd, Sn, Sb, Pb, Hg, and MeHg are shown in mg/kg. For Cr, Cu, Zn, and Se, the regulatory maximum limits and mean daily heavy metal intake amounts from shark meat are shown in mg/day. The regulatory limit of Fe has not been specified. Red box: mean value exceeding the regulatory maximum limit. Yellow box: data that exceed the regulatory maximum limit but the mean value does not. Green box: every data is under the regulatory maximum limit. a: DOH [54]; b: DOH [53]; c: CODEX [52]; d: SCF [59]; e: FAO [56]; f: JECFA [57]; g: EU [55]; h: KFDA [51]; i: UNEP [58].

The regulatory limits of As, Cd, Sn, Sb, Pb, Hg, and MeHg concentrations are directly compared in Table 5. Because Hg and MeHg concentrations are important criteria for determining food safety, various regulatory bodies have presented maximum regulatory levels, and most have reported 1 mg/kg as the limit value [51–57]; however, the Japanese Health Authority has suggested stricter reference value, allowing up to 0.4 mg/kg total Hg and 0.3 mg/kg MeHg [58]. Even with the most stringent standards, the average concentration of Hg and MeHg in all 25 sharks was below the limit value. Almost all 25 sharks showed lower concentrations of Hg and MeHg than the regulatory maximum limits, except for two sharks that had the longest TBL and largest BW (SNU-MO-0006 and SNU-MO-0001). These two sharks had Hg concentrations of 268% and 102% of the maximum limit concentrations, respectively, and both were *C*. *brachyurus* species. Furthermore, only one shark (SNU-MO-0006), which had the longest TBL and largest BW, exceeded the limit for the MeHg concentration (297% of the limit value). In contrast, the As concentrations of all shark species, except for *I*. *oxyrinchus*, exceeded the regulatory limit value. The average concentration was less than the regulatory limit in *I*. *oxyrinchus*, but was fairly close to the limit value. The Average As concentration in all 25 sharks was 7.71 mg/kg, which was more than twice the regulatory level. The concentrations of other heavy metals (Sb, Pb, Cd, and Sn) were within the safe range in all 25 sharks.

For Cr, Cu, Zn, and Se, the regulatory maximum limits were indicated in mg/day [59]; thus, the measured concentrations were converted to mean daily intake amounts of heavy metals from shark meat (mg/day) by multiplying the average amount of seafood ingested by Koreans (79.6 g/day) for each concentration, and these values were then compared with the regulatory limits [60]. The concentrations of all four heavy metals were under the regulatory maximum limits for all shark species (Table 5).

### Food safety of shark meat and the need for shark meat consumption guideline

Shark meat is concluded as not safe to be used as a food source due to the Hg, MeHg, and As concentrations, even though concentrations of Cr, Cu, Zn, Se, Cd, Sn, Sb, and Pb were lower than the regulatory limits (Table 5).

Hg toxicity due to overexposure results in serious clinical problems including neurotoxicity, cardiovascular toxicity, and embryotoxicity in pregnancy, of which Minamata disease is representative. MeHg is one of the organic Hg species and it causes secondary structural change to DNA and RNA and also protein structural change by binding to thiol groups [23]. Since MeHg has a good absorption rate (90%) and long retention time, caution should be exercised when consuming fish meat. According to various regulations for food safety, as discussed above, almost every shark showed Hg and MeHg concentration belonging to the safety zone, except for 2 and 1 individual sharks, respectively.

Even though Hg concentration itself seldom exceeded the regulatory limits in this study, shark meat consumption should still be considered carefully due to various reasons. Previous studies found that combined exposure to Hg and other heavy metals including Al, Cu, Pb, Cd, and Mn poses a synergetic health risk and that shark meat consumption can easily expose an individual to this health risk [61, 62]. Furthermore, excess Hg concentration can be predicted in a copper shark with over 25 kg BW and 130 cm TBL based on the positive relationship found in this study. Since meat from a copper shark over the predicted size will not be appropriate as a food source, the predicted value can be used as a guideline for consumers to choose more safe copper shark meat and for using different shark species as meat. Since Hg concentration also differs from organ to organ, and shark liver, fin, and intestine are also used as food sources, guidelines for different organs should also be prepared [9, 20].

The need for a more detailed guideline for shark meat safety also exists for As, which showed excess concentration in almost every shark examined. Arsenic toxicity results in various manifestations such as skin lesions, chronic lung diseases, liver diseases, and vascular diseases. In addition, since As inhibits mitochondrial respiration, which leads to oxidative stress and successive cell injury, death, and even cancer, its excessive accumulation must be avoided [63]. In this study, As showed significantly higher concentration in benthic sharks than in pelagic sharks, and in fact, all shark species except *I*. *oxyrinchus* showed regulatory limit exceedance. As the concentration of As varies according to the habitat and species, it is necessary to study the As concentration on the basis of a more diversified shark species and propose a guideline to prevent excessive accumulation through shark meat ingestion.

## Conclusions

Sharks are vulnerable to bioaccumulation of various substances including heavy metals since they are apex predators. In this study, five shark species showed As concentrations that exceeded the regulatory maximum limit, whereas almost all Hg and MeHg concentrations were below the regulatory limits. This was likely because the sampled sharks were juveniles, and Hg and MeHg levels showed strong positive correlations with TBL, BW, and age. Because a *C*. *brachyurus* individual of over 25 kg in BW or 130 cm in TBL is likely to have Hg concentrations exceeding the regulatory maximum limit, avoiding consumption of such adult *C*. *brachyurus* can reduce the risk of Hg toxicity. The average concentrations of other heavy metals (Cr, Fe, Cu, Zn, Se, Cd, Sn, Sb, and Pb) were all in the safe range. Despite this, caution should be exercised when consuming shark meat owing to the As, Hg and MeHg concentrations. Since shark meat is being consumed globally, especially in Asian countries, detailed guidelines should further be prepared for safe consumption based on extensive studies of heavy metals in different shark species.

## Supporting information

S1 Table Shapiro-Wilk test & Bartlett’s test results before transformation. Normality assumption and homogeneity of variance-covariance assumption are not all satisfied. All values were rounded to the fourth decimal place.
(DOCX)

S2 Table Estimated transformation parameters for multivariate Box-Cox transformation. Estimated power parameters were replaced by simple power parameters, i.e., 0, ±0.25, and ±0.5.
(DOCX)

S3 Table Shapiro-Wilk test & Bartlett’s test results after transformation. Normality assumption and homogeneity of variance-covariance assumption are satisfied. Even though a few samples tested as non-normal or with slightly inequality of variance, MANOVA is fairly robust to deviations from normality and homoscedasticity. All values were rounded to the fourth decimal place.
(DOCX)

S4 Table Mardia’s multivariate normality (MVN) test & Henze-Zirkler’s MVN test after transformation. Multivariate normality is satisfied. All values were rounded to the third decimal place.
(DOCX)

S5 Table Heavy metal concentrations and biological data of the sampled sharks. Concentrations under detection limit (DL) are marked as <DL. All concentrations are wet weight (ww) concentrations. F: female; M: male.
(DOCX)

S1 Fig Multivariate regression graph. A–K: Multivariate linear regression between BW, TBL, girth and heavy metal concentrations in every 25 sharks. L–O: Multivariate linear regression between BW, TBL and heavy metal concentrations in *C*. *brachyurus*. All A–O showed significant correlation (p<0.05).
(TIF)
