# Supplementary material for: Heavy metal accumulation in and food safety of shark meat from Jeju island, Republic of Korea
Source: PLoS One. 2019 Mar 13;14(3):e0212410. doi: 10.1371/journal.pone.0212410 (PMC6415793; doi:10.1371/journal.pone.0212410)
Supplement: S1 Table — Normality assumption and homogeneity of variance-covariance assumption are not all satisfied. All values were rounded to the fourth decimal place. (DOCX) [file pone.0212410.s001.docx]

**Supplementary materials**

Heavy metal accumulation in and food safety of shark meat from Jeju Island, Republic of Korea

Sang Wha KIM^1^, Se Jin HAN^1^, Yonggab Kim^2^, Jin Woo JUN^3^, Sib Sankar GIRI^1^, Cheng CHI^4^, Saekil YUN^1^, Hyoun Joong KIM^1^, Sang Guen KIM^1^, Jeong Woo KANG^1^, Jun KWON^1^, Woo Taek OH^1^, Jehyun CHA^5^, Seunghee HAN^6^, Byeong Chun LEE^7^, Taesung Park^2^, Byung Yeop KIM^8,*^, and Se Chang PARK^1,*^

^1^Laboratory of Aquatic Biomedicine, College of Veterinary Medicine and Research Institute for Veterinary Science, Seoul National University, Seoul, Republic of Korea

^2^Department of Statistics, College of Natural Sciences, Seoul National University, Seoul, Republic of Korea

^3^Department of Aquaculture, Korea National College of Agriculture and Fisheries, Jeonju, Republic of Korea

^4^Laboratory of Aquatic Nutrition and Ecology, College of Animal Science and Technology, Nanjing Agricultural University, Nanjing, China

^5^School of Mechanical Engineering, Hanyang University, Seoul, Republic of Korea

^6^School of Earth Sciences and Environmental Engineering, Gwangju Institute of Science and Technology, Gwangju, Republic of Korea

^7^Department of Theriogenology and Biotechnology, College of Veterinary Medicine, Seoul National University, Seoul, Republic of Korea

^8^Department of Marine Industry and Maritime Police, College of Ocean Science, Jeju National University, Jeju, Republic of Korea

* Corresponding author

E-mail: kimby@jejunu.ac.kr (BYK)

E-mail: parksec@snu.ac.kr (SCP)

**SUPPLEMENTARY MATERIALS**

**Table 1. Shapiro-Wilk test & Bartlett’s test results before transformation.** Normality assumption and homogeneity of variance-covariance assumption are not all satisfied. All values were rounded to the fourth decimal place.

| Tests | Variables | Fe | Cu | Zn | As | Se | Hg | MeHg |
| --- | --- | --- | --- | --- | --- | --- | --- | --- |
| *All sharks* | | | | | | | | |
| Shapiro-Wilk test | Species | 0.0471 | 0.0002 | 0.0003 | 0.0057 | 7.53e-08 | 1.36e-06 | 1.17e-07 |
|  | Sex | 0.0019 | 4.30e-07 | 0.0110 | 0.0326 | 3.79e-07 | 3.70e-06 | 4.89e-07 |
|  | Habitat | 0.0028 | 3.95e-06 | 9.75e-05 | 0.0792 | 6.59e-08 | 1.21e-06 | 9.85e-08 |
| Bartlett’s test | Species | 0.3350 | 0.0116 | 0.0173 | 0.0550 | 0.0132 | 0.0201 | 0.0038 |
|  | Sex | 0.8099 | 0.7985 | 0.0278 | 0.9001 | 0.0001 | 0.0018 | 0.0001 |
|  | Habitat | 0.4903 | 0.0003 | 0.8884 | 0.1638 | 0.0171 | 0.0013 | 0.0001 |
| *Carcharhinus brachyurus* | | | | | | | | |
| Shapiro-Wilk test | Sex | 0.0069 | 0.0017 | 0.1142 | 0.3260 | 5.66e-05 | 0.0068 | 0.0010 |
| Bartlett’s test | Sex | 0.6372 | 3.06e-07 | 0.0074 | 0.0395 | 0.0170 | 0.0033 | 0.0004 |
