# Supplementary material for: Heavy metal accumulation in and food safety of shark meat from Jeju island, Republic of Korea
Source: PLoS One. 2019 Mar 13;14(3):e0212410. doi: 10.1371/journal.pone.0212410 (PMC6415793; doi:10.1371/journal.pone.0212410)
Supplement: S2 Table — Estimated power parameters were replaced by simple power parameters, i.e., 0, ±0.25, and ±0.5. (DOCX) [file pone.0212410.s002.docx]

**Supplementary materials**

Heavy metal accumulation in and food safety of shark meat from Jeju Island, Republic of Korea

Sang Wha KIM^1^, Se Jin HAN^1^, Yonggab Kim^2^, Jin Woo JUN^3^, Sib Sankar GIRI^1^, Cheng CHI^4^, Saekil YUN^1^, Hyoun Joong KIM^1^, Sang Guen KIM^1^, Jeong Woo KANG^1^, Jun KWON^1^, Woo Taek OH^1^, Jehyun CHA^5^, Seunghee HAN^6^, Byeong Chun LEE^7^, Taesung Park^2^, Byung Yeop KIM^8,*^, and Se Chang PARK^1,*^

^1^Laboratory of Aquatic Biomedicine, College of Veterinary Medicine and Research Institute for Veterinary Science, Seoul National University, Seoul, Republic of Korea

^2^Department of Statistics, College of Natural Sciences, Seoul National University, Seoul, Republic of Korea

^3^Department of Aquaculture, Korea National College of Agriculture and Fisheries, Jeonju, Republic of Korea

^4^Laboratory of Aquatic Nutrition and Ecology, College of Animal Science and Technology, Nanjing Agricultural University, Nanjing, China

^5^School of Mechanical Engineering, Hanyang University, Seoul, Republic of Korea

^6^School of Earth Sciences and Environmental Engineering, Gwangju Institute of Science and Technology, Gwangju, Republic of Korea

^7^Department of Theriogenology and Biotechnology, College of Veterinary Medicine, Seoul National University, Seoul, Republic of Korea

^8^Department of Marine Industry and Maritime Police, College of Ocean Science, Jeju National University, Jeju, Republic of Korea

* Corresponding author

E-mail: kimby@jejunu.ac.kr (BYK)

E-mail: parksec@snu.ac.kr (SCP)

**SUPPLEMENTARY MATERIALS**

**Table 2. Estimated transformation parameters for multivariate Box-Cox transformation.** Variables were replaced by a simple power transformation after estimation.

| Variables | Fe | Cu | Zn | As | Se | Hg | MeHg |
| --- | --- | --- | --- | --- | --- | --- | --- |
| *All sharks* | | | | | | | |
| Species | 0.25 | 0.25 | -0.5 | 0.25 | 0 | -0.25 | -0.25 |
| Sex | 0.25 | 0.25 | -0.25 | 0.25 | 0 | -0.25 | -0.25 |
| Habitat | 0.25 | 0.25 | -0.25 | 0.25 | 0 | -0.25 | -0.25 |
| *Copper shark* | | | | | | | |
| Sex | 0.25 | 0.25 | -0.25 | -0.5 | -0.25 | -0.25 | -0.25 |
