# Supplementary material for: Heavy metal accumulation in and food safety of shark meat from Jeju island, Republic of Korea
Source: PLoS One. 2019 Mar 13;14(3):e0212410. doi: 10.1371/journal.pone.0212410 (PMC6415793; doi:10.1371/journal.pone.0212410)
Supplement: S3 Table — Normality assumption and homogeneity of variance-covariance assumption are satisfied. Even though a few samples tested as non-normal or with slightly inequality of variance, MANOVA is fairly robust to deviations from normality and homoscedasticity. All values were rounded to the fourth decimal place. (DOCX) [file pone.0212410.s003.docx]

**Supplementary materials**

Heavy metal accumulation in and food safety of shark meat from Jeju Island, Republic of Korea

Sang Wha KIM^1^, Se Jin HAN^1^, Yonggab Kim^2^, Jin Woo JUN^3^, Sib Sankar GIRI^1^, Cheng CHI^4^, Saekil YUN^1^, Hyoun Joong KIM^1^, Sang Guen KIM^1^, Jeong Woo KANG^1^, Jun KWON^1^, Woo Taek OH^1^, Jehyun CHA^5^, Seunghee HAN^6^, Byeong Chun LEE^7^, Taesung Park^2^, Byung Yeop KIM^8,*^, and Se Chang PARK^1,*^

^1^Laboratory of Aquatic Biomedicine, College of Veterinary Medicine and Research Institute for Veterinary Science, Seoul National University, Seoul, Republic of Korea

^2^Department of Statistics, College of Natural Sciences, Seoul National University, Seoul, Republic of Korea

^3^Department of Aquaculture, Korea National College of Agriculture and Fisheries, Jeonju, Republic of Korea

^4^Laboratory of Aquatic Nutrition and Ecology, College of Animal Science and Technology, Nanjing Agricultural University, Nanjing, China

^5^School of Mechanical Engineering, Hanyang University, Seoul, Republic of Korea

^6^School of Earth Sciences and Environmental Engineering, Gwangju Institute of Science and Technology, Gwangju, Republic of Korea

^7^Department of Theriogenology and Biotechnology, College of Veterinary Medicine, Seoul National University, Seoul, Republic of Korea

^8^Department of Marine Industry and Maritime Police, College of Ocean Science, Jeju National University, Jeju, Republic of Korea

* Corresponding author

E-mail: kimby@jejunu.ac.kr (BYK)

E-mail: parksec@snu.ac.kr (SCP)

**SUPPLEMENTARY MATERIALS**

**Table 3. Shapiro-Wilk test & Bartlett’s test results after transformation.** Normality assumption and homogeneity of variance-covariance assumption are satisfied. Even though a few samples tested as non-normal or with slightly inequality of variance, MANOVA is fairly robust to deviations from normality and homoscedasticity. All values were rounded to the fourth decimal place.

| Tests | Variables | Fe | Cu | Zn | As | Se | Hg | MeHg |
| --- | --- | --- | --- | --- | --- | --- | --- | --- |
| *All sharks* | | | | | | | | |
| Shapiro-Wilk test | Species | 0.3826 | 0.1606 | 0.7947 | 0.0834 | 0.1242 | 0.5626 | 0.1920 |
|  | Sex | 0.0552 | 0.0146 | 0.7230 | 0.8371 | 0.3933 | 0.7866 | 0.8764 |
|  | Habitat | 0.2087 | 0.0543 | 0.4147 | 0.7111 | 0.2549 | 0.8392 | 0.7926 |
| Bartlett’s test | Species | 0.5023 | 0.2628 | 0.3190 | 0.0747 | 0.0709 | 0.2261 | 0.0977 |
|  | Sex | 0.9543 | 0.2969 | 0.2167 | 0.5445 | 0.2032 | 0.5087 | 0.5299 |
|  | Habitat | 0.2759 | 0.0427 | 0.5710 | 0.9488 | 0.0982 | 0.0918 | 0.0156 |
| *Carcharhinus brachyurus* | | | | | | | | |
| Shapiro-Wilk test | Sex | 0.4399 | 0.0189 | 0.7540 | 0.2873 | 0.8022 | 0.7862 | 0.8913 |
| Bartlett’s test | Sex | 0.1741 | 0.0001 | 0.0464 | 0.3743 | 0.5555 | 0.3750 | 0.3957 |
