# Supplementary material for: Heavy metal accumulation in and food safety of shark meat from Jeju island, Republic of Korea
Source: PLoS One. 2019 Mar 13;14(3):e0212410. doi: 10.1371/journal.pone.0212410 (PMC6415793; doi:10.1371/journal.pone.0212410)
Supplement: S4 Table — Multivariate normality is satisfied. All values were rounded to the third decimal place. (DOCX) [file pone.0212410.s004.docx]

**Supplementary materials**

Heavy metal accumulation in and food safety of shark meat from Jeju Island, Republic of Korea

Sang Wha KIM^1^, Se Jin HAN^1^, Yonggab Kim^2^, Jin Woo JUN^3^, Sib Sankar GIRI^1^, Cheng CHI^4^, Saekil YUN^1^, Hyoun Joong KIM^1^, Sang Guen KIM^1^, Jeong Woo KANG^1^, Jun KWON^1^, Woo Taek OH^1^, Jehyun CHA^5^, Seunghee HAN^6^, Byeong Chun LEE^7^, Taesung Park^2^, Byung Yeop KIM^8,*^, and Se Chang PARK^1,*^

^1^Laboratory of Aquatic Biomedicine, College of Veterinary Medicine and Research Institute for Veterinary Science, Seoul National University, Seoul, Republic of Korea

^2^Department of Statistics, College of Natural Sciences, Seoul National University, Seoul, Republic of Korea

^3^Department of Aquaculture, Korea National College of Agriculture and Fisheries, Jeonju, Republic of Korea

^4^Laboratory of Aquatic Nutrition and Ecology, College of Animal Science and Technology, Nanjing Agricultural University, Nanjing, China

^5^School of Mechanical Engineering, Hanyang University, Seoul, Republic of Korea

^6^School of Earth Sciences and Environmental Engineering, Gwangju Institute of Science and Technology, Gwangju, Republic of Korea

^7^Department of Theriogenology and Biotechnology, College of Veterinary Medicine, Seoul National University, Seoul, Republic of Korea

^8^Department of Marine Industry and Maritime Police, College of Ocean Science, Jeju National University, Jeju, Republic of Korea

* Corresponding author

E-mail: kimby@jejunu.ac.kr (BYK)

E-mail: parksec@snu.ac.kr (SCP)

**SUPPLEMENTARY MATERIALS**

**Table 4. Mardia’s multivariate normality (MVN) test & Henze-Zirkler’s MVN test after transformation.** Multivariate normality is satisfied. All values were rounded to the third decimal place.

| Variables | Mardia’s MVN test (result (p-value)) | | Henze-Zirkler’s MVN test |
| --- | --- | --- | --- |
|  | Mardia skewness | Mardia kurtosis |  |
| *All sharks* | | | |
| Species | Yes (0.284) | Yes (0.963) | Yes (0.401) |
| Sex | Yes (0.705) | Yes (0.365) | Yes (0.367) |
| Habitat | Yes (0.951) | Yes (0.183) | Yes (0.669) |
| *Carcharhinus brachyurus* | | | |
| Sex | Yes (0.772) | Yes (0.093) | Yes (0.293) |
