# Supplementary material for: Heavy metal accumulation in and food safety of shark meat from Jeju island, Republic of Korea
Source: PLoS One. 2019 Mar 13;14(3):e0212410. doi: 10.1371/journal.pone.0212410 (PMC6415793; doi:10.1371/journal.pone.0212410)
Supplement: S5 Table — Concentrations under detection limit (DL) are marked as <DL. All concentrations are wet weight (ww) concentrations. F: female; M: male. (DOCX) [file pone.0212410.s005.docx]

**Supplementary materials**

Heavy metal accumulation in and food safety of shark meat from Jeju Island, Republic of Korea

Sang Wha KIM^1^, Se Jin HAN^1^, Yonggab Kim^2^, Jin Woo JUN^3^, Sib Sankar GIRI^1^, Cheng CHI^4^, Saekil YUN^1^, Hyoun Joong KIM^1^, Sang Guen KIM^1^, Jeong Woo KANG^1^, Jun KWON^1^, Woo Taek OH^1^, Jehyun CHA^5^, Seunghee HAN^6^, Byeong Chun LEE^7^, Taesung Park^2^, Byung Yeop KIM^8,*^, and Se Chang PARK^1,*^

^1^Laboratory of Aquatic Biomedicine, College of Veterinary Medicine and Research Institute for Veterinary Science, Seoul National University, Seoul, Republic of Korea

^2^Department of Statistics, College of Natural Sciences, Seoul National University, Seoul, Republic of Korea

^3^Department of Aquaculture, Korea National College of Agriculture and Fisheries, Jeonju, Republic of Korea

^4^Laboratory of Aquatic Nutrition and Ecology, College of Animal Science and Technology, Nanjing Agricultural University, Nanjing, China

^5^School of Mechanical Engineering, Hanyang University, Seoul, Republic of Korea

^6^School of Earth Sciences and Environmental Engineering, Gwangju Institute of Science and Technology, Gwangju, Republic of Korea

^7^Department of Theriogenology and Biotechnology, College of Veterinary Medicine, Seoul National University, Seoul, Republic of Korea

^8^Department of Marine Industry and Maritime Police, College of Ocean Science, Jeju National University, Jeju, Republic of Korea

* Corresponding author

E-mail: kimby@jejunu.ac.kr (BYK)

E-mail: parksec@snu.ac.kr (SCP)

**SUPPLEMENTARY MATERIALS**

**Table 5. Heavy metal concentrations and biological data of the sampled sharks.** Concentrations under detection limit (DL) are marked as <DL. All concentrations are wet weight (ww) concentrations. F: female; M: male.

| ID | Species | Sampling location (market) | Heavy metal concentration (mg/kg, ww) | | | | | | | | | | | |  | Biologic data | | | | | |
| --- | --- | --- | --- | --- | --- | --- | --- | --- | --- | --- | --- | --- | --- | --- | --- | --- | --- | --- | --- | --- | --- |
|  |  |  | Cr | Fe | Cu | Zn | As | Se | Cd | Sn | Sb | Pb | THg | MeHg |  | Age | TBL (cm) | BW (kg) | Girth (cm) | Sex | Habitat |
| SNU-MO-0001 | *Carcharhinus brachyurus* | Moseulpo | < DL | 26.7147 | 0.5546 | 22.8517 | 15.1225 | 0.5799 | < DL | < DL | < DL | 0.0761 | 0.4089 | 0.2425 |  | 4 | 147 | - | 67 | F | pelagic |
| SNU-MO-0002 | *Carcharhinus brachyurus* | Moseulpo | < DL | 4.6736 | 0.3288 | 4.4662 | 11.1706 | 3.2649 | < DL | < DL | < DL | 0.0835 | 0.2944 | 0.2026 |  | 0 | 100.5 | - | 42 | F | pelagic |
| SNU-MO-0003 | *Carcharhinus brachyurus* | Moseulpo | < DL | < DL | 0.8858 | 7.1427 | 5.4523 | 0.6352 | < DL | 0.2082 | < DL | 0.06 | 0.0665 | 0.0337 |  | 2 | 87 | - | 38 | F | pelagic |
| SNU-MO-0004 | *Carcharhinus brachyurus* | Moseulpo | < DL | 8.2137 | 0.4604 | 13.7624 | 10.4339 | 0.519 | < DL | 0.0351 | < DL | 0.0195 | 0.0963 | 0.0598 |  | 2 | 114 | 9 | 50.5 | F | pelagic |
| SNU-MO-0005 | *Carcharhinus brachyurus* | Moseulpo | < DL | 0.9725 | 0.5482 | 8.7583 | 10.3842 | 0.576 | < DL | < DL | < DL | 0.0217 | 0.1105 | 0.0692 |  | 3 | 109.8 | 8.5 | 45 | F | pelagic |
| SNU-MO-0006 | *Carcharhinus brachyurus* | Moseulpo | 0.1648 | 11.1677 | 1.0163 | 7.3183 | 6.7375 | 0.31 | < DL | 0.0314 | < DL | 0.1962 | 1.0706 | 0.8921 |  | - | 190 | 45 | - | M | pelagic |
| SNU-MO-0007 | *Isurus oxyrinchus* | Moseulpo | < DL | 12.2207 | 7.7498 | 4.3619 | 1.3043 | 0.4194 | < DL | 0.0165 | < DL | 0.0429 | 0.2215 | 0.1322 |  | 1 | 110 | 7.5 | 54 | F | pelagic |
| SNU-MO-0008 | *Carcharhinus obscurus* | Moseulpo | < DL | 2.2459 | 0.1323 | 5.3205 | 8.0156 | 0.3169 | < DL | < DL | < DL | 0.0383 | 0.2713 | 0.1639 |  | 0 | 114 | 7.9 | 52 | M | pelagic |
| SNU-MO-0009 | *Carcharhinus obscurus* | Moseulpo | 0.7492 | 23.0075 | 5.3178 | 10.9969 | 7.6799 | 0.3731 | < DL | 0.1822 | < DL | 0.1293 | 0.2400 | 0.1667 |  | 0 | 118 | 9 | 54 | F | pelagic |
| SNU-MO-0010 | *Isurus oxyrinchus* | Moseulpo | < DL | 14.7325 | 1.1469 | 2.0948 | 2.8112 | 0.2931 | < DL | 0.1302 | < DL | 0.0687 | 0.3266 | 0.2399 |  | - | 137 | 17.7 | - | M | pelagic |
| SNU-MO-0011 | *Cephaloscyllium umbratile* | Hallim | 0.1588 | 5.657 | 0.5731 | 13.5636 | 8.0886 | 0.2401 | < DL | < DL | 0.0774 | 0.0697 | 0.1806 | 0.1094 |  | - | 63 | - | - | M | benthic |
| SNU-MO-0012 | *Carcharhinus brachyurus* | Moseulpo | 0.0032 | 2.9276 | 8.9158 | 4.4975 | 4.0259 | 0.7248 | < DL | 0.0063 | 0.0443 | 0.0063 | 0.1720 | 0.1111 |  | 0 | 68 | 1.65 | 25 | M | pelagic |
| SNU-MO-0013 | *Carcharhinus brachyurus* | Moseulpo | 0.083 | 3.2612 | 1.0627 | 13.8818 | 6.9973 | 0.2159 | < DL | 0.0199 | 0.0465 | 0.0166 | 0.1491 | 0.0748 |  | 3 | 143 | 19.5 | 60 | F | pelagic |
| SNU-MO-0014 | *Carcharhinus brachyurus* | Moseulpo | < DL | 6.9166 | < DL | 4.157 | 4.5788 | 0.1288 | < DL | 0.0354 | < DL | 0.0354 | 0.0983 | 0.0628 |  | 1 | 92 | 4.5 | - | M | pelagic |
| SNU-MO-0015 | *Carcharhinus brachyurus* | Moseulpo | 0.011 | 20.6689 | 3.6917 | 5.0847 | 5.8473 | 0.1613 | < DL | 0.044 | < DL | 0.0403 | 0.1168 | 0.0620 |  | 2 | 118 | 9.2 | 47.5 | M | pelagic |
| SNU-MO-0016 | *Carcharhinus brachyurus* | Moseulpo | 0.103 | 3.1122 | 0.5187 | 2.3799 | 4.9429 | 0.0687 | < DL | < DL | < DL | 0.0038 | 0.116586 | 0.072 |  | 2 | 121 | 12 | 51 | F | pelagic |
| SNU-MO-0017 | *Carcharhinus brachyurus* | Moseulpo | < DL | 7.3542 | 0.7387 | 3.6387 | 3.924 | 0.0841 | < DL | 0.0037 | < DL | < DL | 0.373888 | 0.208 |  | 7 | 165 | 26 | 69 | M | pelagic |
| SNU-MO-0018 | *Carcharhinus brachyurus* | Moseulpo | 0.0425 | 2.0243 | 0.2477 | 2.1765 | 4.4131 | 0.1557 | < DL | < DL | < DL | < DL | 0.153162 | 0.102 |  | 3 | 140 | 16.6 | 56.5 | F | pelagic |
| SNU-MO-0019 | *Carcharhinus brachyurus* | Moseulpo | 0.5674 | 12.8523 | 1.1071 | 3.609 | 5.6972 | 0.2719 | < DL | < DL | < DL | < DL | 0.236728 | 0.117 |  | 5 | - | - | 57.5 | - | pelagic |
| SNU-MO-0020 | *Mustelus manazo* | Hallim | 0.1247 | 4.0576 | 0.6725 | 2.0363 | 15.0213 | 0.4496 | < DL | < DL | < DL | 0.0113 | 0.148082 | 0.089 |  | 2 | 84 | - | 25.5 | F | benthic |
| SNU-MO-0021 | *Triakis scyllium* | Hallim | 0.7698 | 25.4804 | 0.2694 | 4.1184 | 8.718 | 0.7621 | < DL | < DL | < DL | 0.0231 | 0.148082 | 0.086 |  | 2 | 74 | 1.7 | 26.5 | M | benthic |
| SNU-MO-0022 | *Mustelus manazo* | Hallim | 0.0138 | < DL | 0.1797 | 2.149 | 20.1565 | 0.3731 | < DL | < DL | < DL | < DL | 0.092202 | 0.066 |  | - | 52 | 0.5 | 16.5 | M | benthic |
| SNU-MO-0023 | *Carcharhinus brachyurus* | Moseulpo | 0.0676 | 12.3939 | 0.823 | 4.938 | 4.9718 | 0.0789 | < DL | < DL | < DL | 0.0188 | 0.213868 | 0.131 |  | 3 | 141 | 16.7 | 56 | F | pelagic |
| SNU-MO-0024 | *Triakis scyllium* | Hallim | 0.1701 | 3.3286 | 0.1314 | 2.9768 | 7.9794 | 0.4523 | < DL | < DL | < DL | 0.0232 | 0.138176 | 0.087 |  | - | 69.5 | - | 26 | M | benthic |
| SNU-MO-0025 | *Triakis scyllium* | Hallim | < DL | 1.1079 | 0.1443 | 3.1325 | 8.2584 | 0.7061 | < DL | < DL | < DL | < DL | 0.105156 | 0.049 |  | - | 66.5 | - | 26.5 | M | benthic |
