# Supplementary figures and images for: Heavy metal accumulation in and food safety of shark meat from Jeju island, Republic of Korea
Source: PLoS One. 2019 Mar 13;14(3):e0212410. doi: 10.1371/journal.pone.0212410 (PMC6415793; doi:10.1371/journal.pone.0212410)

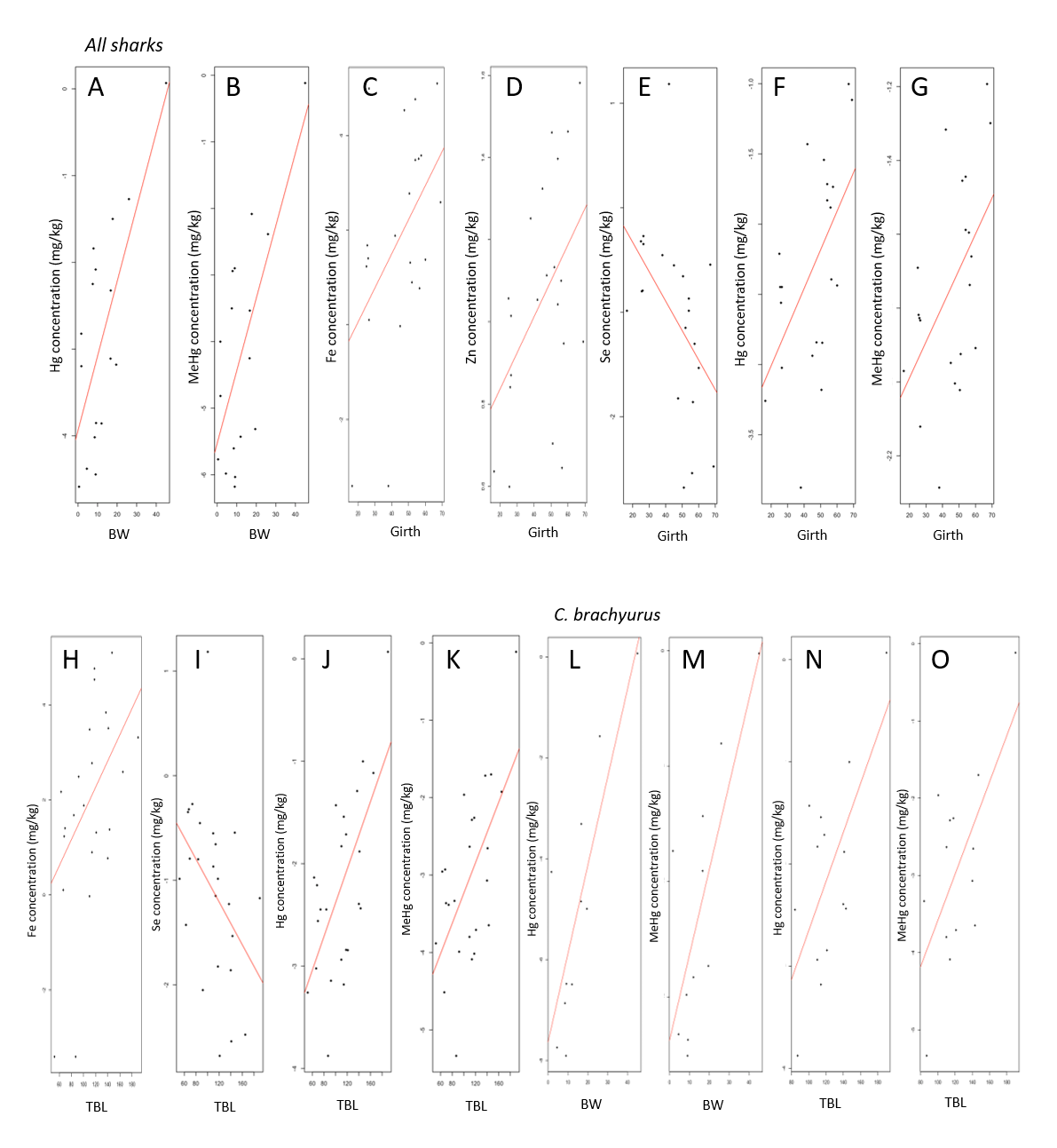

Supplement: S1 Fig — A–K: Multivariate linear regression between BW, TBL, girth and heavy metal concentrations in every 25 sharks. L–O: Multivariate linear regression between BW, TBL and heavy metal concentrations in C. brachyurus. All A–O showed significant correlation (p<0.05). (TIF) [file pone.0212410.s006.tif]
